# Supplementary material for: Effect of Selenium Deficiency on the Development of Overt Hepatic Encephalopathy in Patients with Chronic Liver Disease
Source: J Clin Med. 2023 Apr 14;12(8):2869. doi: 10.3390/jcm12082869 (PMC10143189; doi:10.3390/jcm12082869)

Figure S1. Cumulative incidence of OHE in patients with and without selenium deficiency. OHE probability was estimated using the Kaplan-Meier method and compared between the two groups using the log-rank test. OHE, overt hepatic encephalopathy.

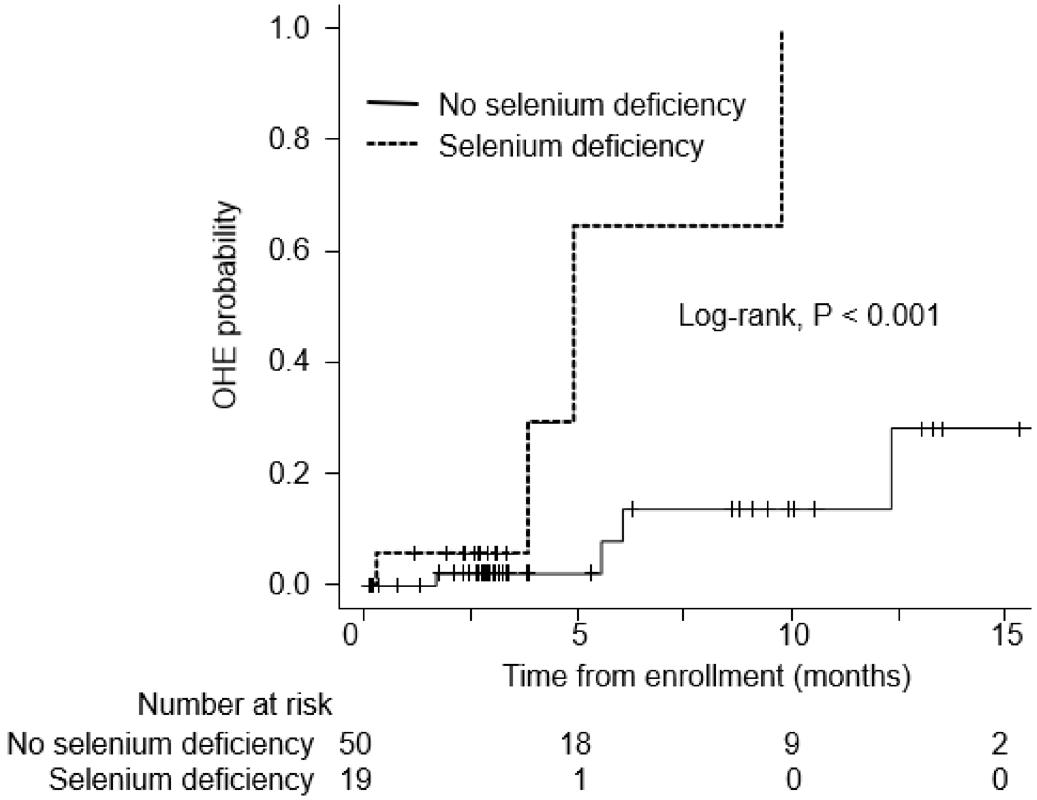

Supplement: Supplementary file 1 [file jcm-12-02869-s001.zip › jcm-2322074-supplementary_Figure S1.pdf]
