# Supplementary material for: Effect of Selenium Deficiency on the Development of Overt Hepatic Encephalopathy in Patients with Chronic Liver Disease
Source: J Clin Med. 2023 Apr 14;12(8):2869. doi: 10.3390/jcm12082869 (PMC10143189; doi:10.3390/jcm12082869)
Supplement: Supplementary file 1 [file jcm-12-02869-s001.zip › jcm-2322074-supplementary_Table S1.pdf]

Table S1 The calculation methods of the (1) FIB-4 index, (2) ALBI score, and (3) Child-Pugh score

(1) The FIB-4 values were calculated using the following formula: age (years) × aspartate aminotransferase (IU/L) / (platelet counts [10^9/L]) × (alanine aminotransferase [IU/L]$^{1/2}$)[1]

(2) The ALBI score was calculated using the following formula: ($\log_{10}$ bilirubin (μmol/L) × 0.66) + (albumin (g/L) × –0.085). The ALBI grade was determined based on the ALBI score as follows: ≤–2.60 = Grade 1, >–2.60 to ≤–2.27 = Grade 2a, >–2.27 to ≤–1.39 = Grade 2b, >–1.39 = Grade 3 [2].

(3)The Child-Pugh score was scored using five factors (hepatic encephalopathy, ascites, total bilirubin, albumin, and prothrombin time) and rated on a three-point scale (Class A to C)[3]. The sum of the points for each category was used to determine the overall Child-Pugh score, which can range from 5 to 15. Class A corresponds to 5–6 points, Class B to 7–9 points, and Class C to 10–15 points.

**Category**	**1 point**	**2 points**	**3 points**
Hepatic encephalopathy	None	Grade 1–2	Grade >3
Ascites	None	Mild	Moderate/severe
Total bilirubin (mg/dL)	<2	2.0–3.0	>3.0
Albumin (g/dL)	>3.5	2.8–3.5	<2.8
Prothrombin time (%)	>70	40–70	<40

References

1. Vallet-Pichard, A.; Mallet, V.; Nalpas, B.; Verkarre, V.; Nalpas, A.; Dhalluin-Venier, V.; Fontaine, H.; Pol, S. FIB-4: an inexpensive and accurate marker of fibrosis in HCV infection. comparison with liver biopsy and fibrotest. *Hepatology* **2007**, *46*, 32-36, doi:10.1002/hep.21669.
2. Johnson, P.J.; Berhane, S.; Kagebayashi, C.; Satomura, S.; Teng, M.; Reeves, H.L.; O'Beirne, J.; Fox, R.; Skowronska, A.; Palmer, D.; et al. Assessment of liver function in patients with hepatocellular carcinoma: a new evidence-based approach-the ALBI grade. *J Clin Oncol* **2015**, *33*, 550-558, doi:10.1200/jco.2014.57.9151.
3. Pugh, R.N.; Murray-Lyon, I.M.; Dawson, J.L.; Pietroni, M.C.; Williams, R. Transection of the oesophagus for bleeding oesophageal varices. *Br J Surg* **1973**, *60*, 646-649, doi:10.1002/bjs.1800600817.
